# Effects of Echinacea Purpurea Polysaccharides on Growth Performance, Serum Biochemistry, and Intestinal Health of Immunosuppressed Broilers

**DOI:** 10.3390/ani15203036

**Published:** 2025-10-20

**Authors:** Zhiying Zhang, Su Peng, Hyerin Jung, Peining She, Wanqi Li, Yang Xiao, Aiting Shan, Xiaojie Huang, Dayou Shi

**Affiliations:** 1 College of Veterinary Medicine, South China Agricultural University, Guangzhou 510642, China; zzhying17@163.com (Z.Z.); pengsu222@163.com (S.P.); juliusphinx@gmail.com (H.J.); shepeining0815@163.com (P.S.); beckycaca@hotmail.com (W.L.); 18565194514@163.com (Y.X.); 17660630205@163.com (A.S.); 2 College of Animal Science and Technology, Guangxi Vocational University of Agriculture, Nanning 530007, China; fjhxj168@126.com

**Keywords:** Echinacea purpurea polysaccharide, immunosuppression, growth performance, intestinal barrier, serum biochemistry

## Abstract

**Simple Summary:**

Intensive farming is increasingly becoming the prevailing trend in animal production, yet it often compromises immune function and elevates stress levels in poultry. Echinacea purpurea polysaccharide (EPP) has been demonstrated to possess antioxidant, anti-inflammatory, and immune-enhancing properties. This study was designed to investigate the effects of dietary supplementation with different doses of EPP on Mahuang chickens under immunosuppressed conditions. By incorporating EPP into the diet of immunosuppressed Mahuang chickens, we found that EPP supplementation improved growth performance and immune organ indices. Furthermore, it exerted beneficial effects on intestinal morphology, intestinal gene expression, and serum biochemical parameters. These findings provide valuable insights for the potential application of EPP as a dietary supplement in intensive poultry production systems.

**Abstract:**

Echinacea polysaccharide (EPP) is one of the main active ingredients of Echinacea purpurea and has been shown to possess antioxidant, anti-inflammatory, and immune-enhancing activities. The study investigated the effects of supplementing the diet with different doses of EPP on immunosuppressed broilers. A total of 180 one-day-old healthy broilers were randomly assigned to six groups, each with six replicates of five birds. C (control) and CTX (cyclophosphamide) groups received basal diet, while LLEP, LEP, MEP, and HEP groups were supplemented with 100, 200, 400, and 800 mg/kg EPP. On day 7, group C was injected with saline for three consecutive days, whereas the remaining groups received cyclophosphamide (CTX, 80 mg/kg) to induce immunosuppression. Intestine, liver, and serum samples were collected on days 14 and 28 for analysis. The results showed that all EPP-supplemented groups exhibited improved growth performance compared to the CTX group, and the immune organ index increased. Specifically, the MEP group showed an improvement in jejunal morphology, and the LLEP and LEP groups improved ileal morphology. The EPP-added groups had improved ileal morphology The EPP-added group exhibited improved jejunal and ileal intestinal barriers, i.e., Occludin, Claudin1, Claudin2 and MUC2 at different time periods, as well as immune-related markers, at different time points. Furthermore, the MEP and HEP groups showed upregulated Nrf2 gene expressions in the jejunum and ileaum. EPP supplementation reduced MDA contents in serum, liver, and small intestine. The LLEP group effectively increased GSH-Px in serum and liver, while the MEP group effectively increased T-AOC in serum, liver, and small intestine. Meanwhile, compared to the CTX group, the MEP group showed increased ALB levels and all groups supplemented with EPP showed elevated TP levels. In conclusion, EPP ameliorated cyclophosphamide-induced immunosuppression in broilers, with the optimal effect observed at a supplementation level of 400 mg/kg (MEP group).

## 1. Introduction

Poultry meat products, serving as a low-cost source of protein, have experienced a significant expansion in breeding scale in recent years. Currently, the poultry farming industry has reached a peak level of development globally [1]. With the growth of the global poultry sector, intensive farming practices are increasingly becoming the dominant mode of production. However, this intensification has also heightened the susceptibility of poultry to pathogenic hazards and environmental contamination, thereby elevating stress-related risks in birds [2,3]. Immunosuppression, defined as a state of impaired immune function, can be triggered by infections, stressors, or other pressures [3,4]. This immune impairment heightens the host’s sensitivity to pathogens [5]. Cyclophosphamide (CTX), widely utilized in both veterinary and human medicine as an antitumor agent and is commonly employed to induce immunosuppressive models. Immunosuppression refers to the disruption or suppression of the immune system caused by various factors such as viruses, bacteria, and environmental stressors. In broiler production, several immunosuppressive diseases—such as Marek’s disease and avian leukosis—are prevalent and can inhibit immune responses [6]. Ji et al. [6] used CTX to construct an immunosuppressive model for broilers. CTX acts by suppressing both humoral and cellular immunity, thereby inducing immunodeficiency in animals [7,8,9]. Moreover, it has been shown to cause damage to the intestinal mucosal barrier [3].

The intestine represents the largest interface between an organism and the external environment. The stability of the intestinal mucosal barrier is essential for overall host health [10]. Impairment of this epithelial barrier can facilitate the onset of both intestinal and systemic infections [11]. In addition, intestinal integrity plays a critical role in preserving the homeostasis of the intestinal lumen and preventing systemic dissemination of pathogens [12]. The intestinal barrier effectively prevents harmful intestinal substances from entering the circulation. However, disrupting the intestinal barrier may trigger an immune response [13]. Intestinal morphology is another key determinant of organismal health [14], as a complex interdependence exists between structural integrity and intestinal function that affects nutrient absorption and immune responses. Compromised intestinal health adversely affects growth, development, and disease susceptibility [15]. Under intensive farming conditions, intestinal dysfunction is a common pathological outcome that can readily impair poultry health and lead to substantial economic losses [16].

In recent years, extensive research has explored the use of natural plant additives to enhance poultry gut health. Echinacea purpurea (EP), an herbaceous perennial plant in the Asteraceae family, is native to North America, primarily distributed from the eastern and central United States to southern Canada. Historically utilized in the United States since the nineteenth century, it has been employed in the treatment of colds, infections, and bites from venomous insects and snakes [17,18,19,20]. Echinacea extract contains numerous bioactive constituents, including polysaccharides, alkylamides, caffeic acid derivatives, phenylpropanoids, and volatile oils [21]. Among these, Echinacea purpurea polysaccharide (EPP) is a major active component of EP and has been shown to exhibit multiple bioactive properties, such as antioxidant, anti-inflammatory, antimicrobial, immunomodulatory, and anticancer effects [22,23,24]. A study by Li et al. [20] further showed that EPP could alleviate colitis in rats by regulating Th17/Treg balance and inhibiting TLR4/Myd88/NF-κB pathway. Moreover, numerous studies indicate that plant-derived polysaccharides can enhance growth performance, immune organ indices, immunity, and gut health in poultry [25]. However, few studies have addressed the role of EPP in immunosuppression of Mahuang chickens. Mahuang chickens are one of the most popular broiler varieties in South China, with substantial consumption. As early as 2018, the number of Ma Partridge chickens slaughtered annually had already reached between 520 million and 560 million. Moreover, compared with Arbor Acres broilers, Mahuang chickens exhibit superior meat quality, making them more favored in the frozen market [26,27,28].

This experiment investigated the effects of EPP on immunosuppressed broilers. The aim was to evaluate the impact of different EPP concentrations on growth performance, organ indices, serum biochemistry, antioxidant status, and intestinal barrier function in these birds, providing a theoretical basis for future related research.

## 2. Materials and Methods

### 2.1. Animal Ethics

The methodology involving animals in this study strictly adhered to the guidelines and regulations set by the South China Agricultural University Committee (Record No. 2025F310) to ensure ethical and responsible behavior throughout the study.

### 2.2. Preparation of Polysaccharides

In this experiment, reference was made to Li [24] who extracted EPP using hydroalcoholic method. Whole plant of Echinacea which was produced in July 2023 was taken and cut into pieces and soaked in 80% ethanol for 12 h. The filtrate was discarded; the herbs were wrapped in gauze, decocted in pure water, brought to a boil over high heat, maintained at a gentle boil, and decocted twice. The two decoctions were combined and concentrated using a rotary evaporator to achieve a crude-drug concentration of 1 g/mL. The concentrate was then centrifuged at 4500 r/min for 5 min to remove insoluble impurities. The resulting supernatant was subjected to ethanol precipitation, which was repeated three times. Finally, the purified product was freeze-dried to obtain the Echinacea purpurea polysaccharide (EPP) sample. The polysaccharide content of the samples was measured using the phenol-sulfuric acid method [29] and the EPP content was 26.11 mg/g (dry-weight basis).

### 2.3. Animals and Groups

A total of 180 one-day-old healthy Mahuang chickens (Jiangmen Kelang Agricultural Science and Technology Co., Ltd., Guangdong, China) with similar initial body weights were randomly divided into 6 groups, each with 6 replicates of 5 chicks each. Broilers had ad libitum access to feed and water. The experimental groups were control (C), cyclophosphamide (CTX), ultra-low-dose EPP (LLEP), low-dose EPP (LEP), medium-dose EPP (MEP) and high-dose EPP (HEP). The C and CTX groups were fed a basal (control) ration, and the LLEP, LEPP, MEP, and HEP groups were supplemented with 100 mg/kg, 200 mg/kg, 400 mg/kg, and 800 mg/kg of EPP in the basal diet, respectively. The experiment was conducted for a total of 28 days. Starting from day 7, birds in the CTX, LLEP, LEP, MEP, and HEP groups received cyclophosphamide (80 mg/kg) by intrapectoral injection once daily for three consecutive days (08:00–09:00) in the CTX, LLEP, LEP, MEP and HEP groups.

### 2.4. Experimental Diet Design

The feeding trials were conducted at the animal feeding base of South China Agricultural University. The experimental diets were formulated according to the nutritional requirements of Chinese chickens (NY/T33-2004) [30,31], and the dietary composition and nutrient levels are shown in Table S1. All groups were maintained under identical dietary and lighting conditions. The ambient temperature was kept at 35 °C for the first week and then gradually reduced by 3 °C weekly until it reached 26 °C. The relative humidity was maintained at 60% to 80%.

### 2.5. Growth Performance

Average daily gain (ADG, g), average daily feed intake (ADFI, g) and weekly feed intake were recorded. Feed conversion ratio (FCR) was recorded weekly for all groups.

To calculate ADG, amount of weight gained in body during a period was divided by the number of days during that period. To calculate ADFI, amount of feed consumption during the period was divided by the number of days and the number of chickens during that period. And FCR = ADFI/ADG.

### 2.6. Mahuang Chickens Sample Collection

On days 14 and 28 of the trial, body weight and feed weight were recorded, and then one chicken (*n* = 6) from each replicate was randomly selected according to the American Veterinary Medical Association guidelines, and approximately 5 mL of blood was withdrawn from the subwing vein, separated from the serum by centrifugation at 3500 rpm for 10 min at 4 °C, and then stored at −20 °C. Broilers were subsequently euthanized by cervical dislocation. Thymus, spleen, bursa and intestines were removed separately and weighed by trained personnel. Use the following formula for organ index calculations: organ index (mg/g) = spleen, thymus, or bursa weight (mg)/body weight (g). At the same time 2 cm of small intestine was removed and fixed in 10% formalin for histomorphometric examination. In addition to this the collected small intestines were placed in RNase and DNase free tubes and immediately quick-frozen in liquid nitrogen, and then stored at −80 °C for further analysis.

### 2.7. Histopathological Staining

The taken 2 cm samples of small intestine (jejunum and ileum) (6 from each group) were dehydrated and preserved for 72 h, then hyalinized and embedded in paraffin. Transverse sections were fixed on slides, followed by staining with hematoxylin and eosin, (H&E) and finally sealed. The length of villi (VH, villus tip to crypt), and depth of crypt (CD, base of villi to submucosa) of jejunal, using NDP View2 (Hamamatsu Photonics Co., Ltd., Shizuoka Prefecture, Japan) for measurement and analysis of intestinal tissue sections. A total of 10 sets of data were measured for each index, and the mean values were calculated. The ratio of villus length to crypt depth (VH/CD) was calculated as an index to assess the extent of intestinal injury.

### 2.8. Real-Time Quantitative PCR Analysis

Real-time fluorescence quantitative PCR (qPCR) was performed to determine the mRNA expression levels of target genes in the jejunum and ileum. Total RNA was extracted from approximately 30–50 mg of frozen intestinal tissue using an RNA extraction kit (Subiopure Co., Ltd., Guangdong, China). The tissue was homogenized in lysis buffer (TRIzol plus) using a homogenizer, followed by vortexing and incubation at room temperature. Subsequently, 200 µL of chloroform was added, and the mixture was shaken and incubated. After centrifugation at 12,000× *g* for 10 min at 4 °C, the aqueous phase containing RNA was transferred to a new tube. RNA concentration and purity were assessed using a NanoDrop 1000 Spectrophotometer. All RNA samples showed A260/A280 ratios between 1.8 and 2.0, indicating high purity and suitability for reverse transcription. cDNA synthesis was carried out with the HiFiScript All-in-One RT Master Mix for qPCR (CWBIO Co., Ltd., Jiangsu, China). qPCR reactions were conducted on an Analytik Jena qTOWER3/G system using 5× HIFIScript ALL-in-one qRT Master Mix (CWBIO Co., Ltd., Jiangsu, China). β-Actin was used as the internal reference gene, and the specific primer sequences are listed in Table S2 (synthesized by Sangon Biotech, Shanghai, China). Each 5 µL PCR reaction contained 1 µL cDNA (50 ng/µL), 0.2 µL each of forward and reverse primers (10 µM), and 3.6 µL ddH_2_O. The thermal cycling conditions consisted of initial denaturation at 95 °C for 30 s, followed by 40 cycles of 95 °C for 15 s and 60 °C for 30 s. Gene expression levels in the jejunum and ileum were calculated using the 2^−∆∆CT^ method.

### 2.9. Antioxidant Kit Assay

Use of antioxidant kits (Nanjing Jiancheng bioengineering Co., Ltd., Nanjing, China), antioxidant parameters including malondialdehyde (MDA), total antioxidant capacity (T-AOC) and total superoxide dismutase (T-SOD) were tested in duplicate in the jejunum and ileum according to the manufacturer’s instructions; Antioxidant indices in serum and liver including glutathione peroxide (GSH-Px), MDA, T-AOC, and T-SOD were tested in duplicate. Measurements were made at the indicated wavelengths using an enzyme marker.

GSH-Px activity was measured using a commercial assay kit (A005-1, Nanjing Jiancheng Bioengineering Institute, Nanjing, China) according to the manufacturer’s instructions. The assay principle is that GSH-Px catalyzes the reaction of hydrogen peroxide (H_2_O_2_) with reduced glutathione (GSH) to produce water and oxidized glutathione (GSSG). Enzyme activity, determined by the rate of GSH consumption during the reaction, was quantified by measuring the absorbance at 412 nm using a microplate reader.

T-AOC was assessed using a commercial assay kit (A015-1, Nanjing Jiancheng Bioengineering Institute, Nanjing, China) following the manufacturer’s instructions. The assay is based on the principle that antioxidants in a sample can reduce Fe^3+^ to Fe^2+^. The resulting Fe^2+^ ions form a stable chromogenic complex with phenanthroline derivatives, and the intensity of this complex, measured at 520 nm using a microplate reader, is proportional to the T-AOC. The value was then calculated using a standard formula.

T-SOD activity was determined using a commercial assay kit (A001-1, Nanjing Jiancheng Bioengineering Institute, Nanjing, China) according to the manufacturer’s instructions. This hydroxylamine method employs a xanthine/xanthine oxidase system to generate superoxide anion radicals (O_2_^−^·), which then oxidize hydroxylamine to form nitrite. The nitrite produces a purple-red color in the presence of a chromogenic agent, and the absorbance at 550 nm was measured with a microplate reader. T-SOD activity in the samples was subsequently calculated using a standard formula.

MDA concentration was determined using a commercial assay kit (A003-1, Nanjing Jiancheng Bioengineering Institute, Nanjing, China) according to the manufacturer’s instructions. The assay employed the thiobarbituric acid (TBA) method, in which MDA reacts with TBA to form a red adduct that has a maximum absorption peak at 532 nm. The absorbance at this wavelength was measured, and the MDA concentration in the samples was calculated using a standard formula.

TP concentration was determined using a commercial assay kit (A045-2, Nanjing Jiancheng Bioengineering Institute, Nanjing, China) according to the manufacturer’s instructions. The assay is based on the Coomassie brilliant blue method, where the binding of dye anions to amino groups (–NH_3_^+^) on protein molecules results in a color change from brown-red to blue. The absorbance of this blue complex was measured at 595 nm, and the protein concentration was calculated using a standard formula.

Prior to analysis, all tissue samples were pre-processed as follows: a precise weight of animal tissue was homogenized with nine volumes of physiological saline (1:9 weight-to-volume ratio) under low-temperature conditions (0–4 °C). The homogenate was centrifuged at 3500 rpm for 10 min, and the resulting supernatant was collected for subsequent assays.

### 2.10. Serum Biochemical Indicators

The collected serum is used to measure biochemically related parameters. Total protein (TP), albumin (ALB) and globulin (GLB) should be used in accordance with the manufacturer’s instructions using the commercial kit (Mindray Co., Ltd., Guangdong, China) for testing.

### 2.11. Statistical Analysis

All data are presented as mean ± standard deviation (SD) from at least three independent experiments. Data were analyzed and processed using SPSS 26 (IBM, Armonk, NY, USA) and Graph Pad Prism 8 (Graph Pad Software Inc., San Diego, CA, USA). One-way analysis of variance and Duncan’s multiple range test were used to determine the differences between groups, and *p* < 0.05 was considered statistically significant.

## 3. Results

### 3.1. Effect of Echinacea Purpurea Polysaccharide on the Body Weight of Immunosuppressed Chickens

As shown in Table 1, at 14 days of age, the ADG in the MEP group was significantly higher than in the CTX group (*p* < 0.001), and no significant difference compared with the C group. Furthermore, the ADFI in the LLEP and MEP groups was significantly lower than in the CTX group (*p* < 0.05). At 21 days of age, ADG in the LEP, MEP, and HEP groups was significantly higher than in the CTX group (*p* < 0.05), with no difference observed between the C group and the treatment groups. Additionally, ADFI in the LEP, MEP, and HEP groups was significantly lower than in the CTX group (*p* < 0.05), but no significant difference was found between the LEP group and the C group. Moreover, the F/G in the EPP-supplemented groups was significantly lower than in the CTX group (*p* < 0.05), with no differences observed between the LEP, MEP, or HEP groups and the C group. At 28 days of age, ADFI in the LEP group was significantly lower than in the CTX group (*p* < 0.05), but not significantly different from that in the C group. Similarly, F/G in the EPP-supplemented groups was significantly lower than in the CTX group (*p* < 0.05), with no significant difference compared to the C group.

### 3.2. Effect of Echinacea Purpurea Polysaccharides on Organ Index in Immunosuppressed Chickens

As shown in Table 2, at 14 days of age, the splenic index in the LEP, MEP, and HEP groups did not differ significantly from that of group C. The Bursal index in the HEP group was significantly higher than that in the CTX group (*p* < 0.05), but did not differ from that in Group C.

### 3.3. Effect of Immunosuppressed Chickens on Intestinal Morphology

**Figure 1:**
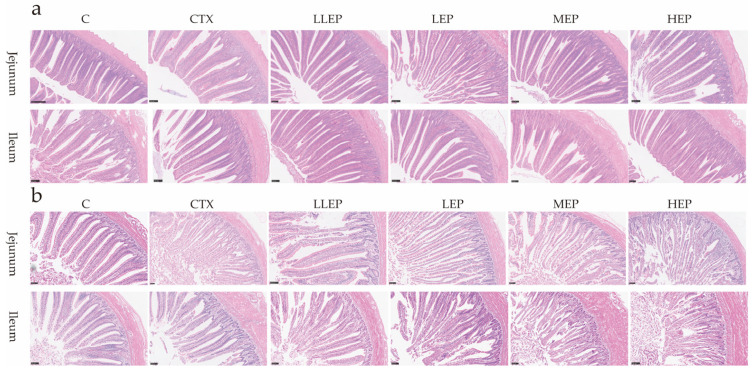
The effect of EPP on intestinal morphology in immunosuppressed chickens (20×, Scale bar = 100 μm). The effect of 14 days old EPP on intestinal morphology in immunosuppressed chickens (**a**); The effect of 28 days old EPP on intestinal morphology in immunosuppressed chickens (**b**). Abbreviations: C, unimmunosuppressed broilers fed a basic diet; CTX, immunosuppressed broilers fed a basic diet; LLEP, for immunosuppressed broilers, 100 mg/kg of EPP were added to the basic diet; LEP, for immunosuppressed broilers, 200 mg/kg of EPP were added to the basic diet; MEP, for immunosuppressed broilers, 400 mg/kg of EPP were added to the basic diet; and HEP, for immunosuppressed broilers, 800 mg/kg of EPP were added to the basic diet.

As shown in Table 3, at 14 days of age, the villus height (VH) in the jejunum of the MEP group was significantly higher than that of the CTX group (*p* < 0.05). At 28 days of age, in the jejunum, VH was significantly higher in all EPP-supplemented groups compared to both the CTX and C groups, while crypt depth (CD) was significantly higher in the HEP group compared to the CTX group (*p* < 0.05). In the ileum, VH was significantly higher in the LLEP and LEP groups compared to the CTX group (*p* < 0.05), with the LLEP group also showing a significant increase over the C group (*p* < 0.05). Figure 1 also shows the intestinal villi from the different groups.

### 3.4. Effect of Echinacea Purpurea Polysaccharides on Gene Expression in Immunosuppressed Chickens

**Figure 2:**
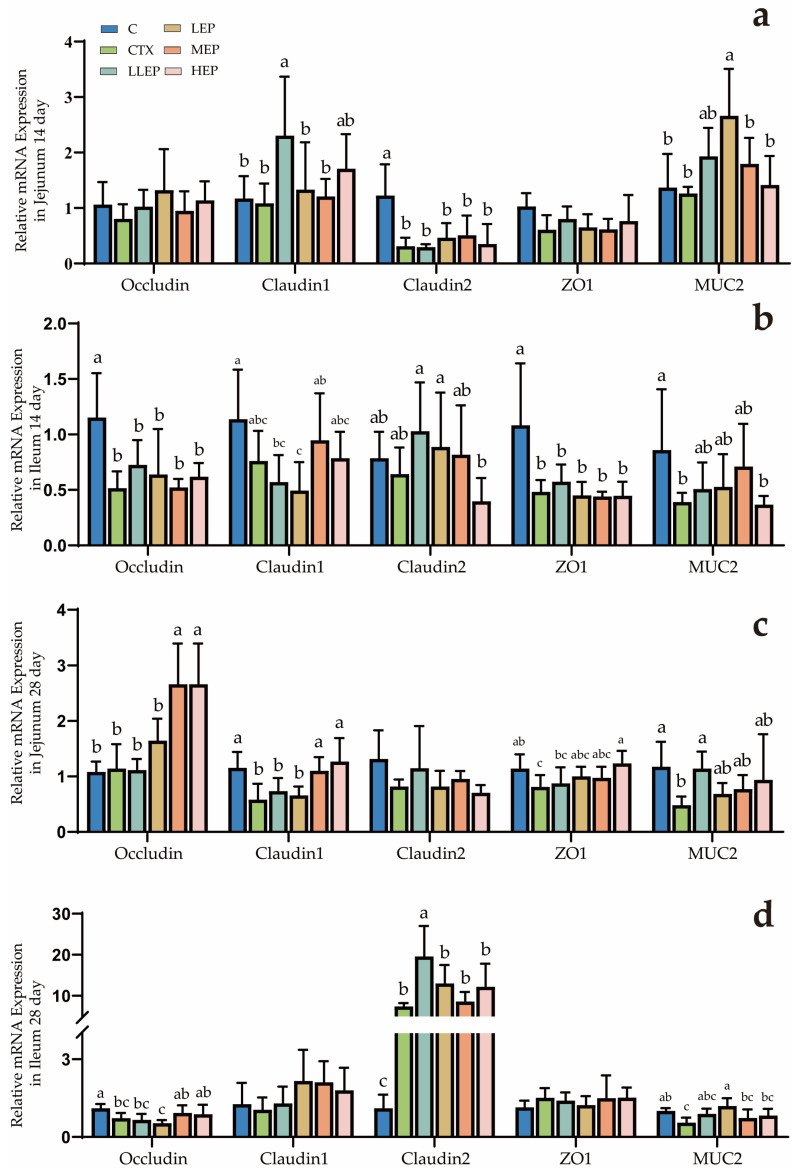
Effect of dietary addition of EPP on the expression of genes related to the intestinal barrier in immunosuppressed chickens. (**a**): Effect of dietary addition of EPP on the expression of genes related to the jejunum barrier in immunosuppressed chickens at 14 days; (**b**): Effect of dietary addition of EPP on the expression of genes related to the ileum barrier in immunosuppressed chickens at 14 days; (**c**): Effect of dietary addition of EPP on the expression of genes related to the jejunum barrier in immunosuppressed chickens at 28 days; (**d**): Effect of dietary addition of EPP on the expression of genes related to the ileum barrier in immunosuppressed chickens at 28 days. ^abc^ Values within a column followed by different superscripts are significantly different. *p* < 0.05. Abbreviations: C, unimmunosuppressed broilers fed a basic diet; CTX, immunosuppressed broilers fed a basic diet; LLEP, for immunosuppressed broilers, 100 mg/kg of EPP were added to the basic diet.; LEP, for immunosuppressed broilers, 200 mg/kg of EPP were added to the basic diet; MEP, for immunosuppressed broilers, 400 mg/kg of EPP were added to the basic diet; and HEP, for immunosuppressed broilers, 800 mg/kg of EPP were added to the basic diet.

As shown in Figure 2, at 14 days of age, the LLEP group increased the expression of Claudin-1 (*p* = 0.040) in the jejunum, while the LEP group elevated the expression of MUC2 (*p* = 0.004) in the jejunum. At 28 days of age, the MEP and HEP groups increased the expression of Occludin (*p* < 0.001) and Claudin-1 (*p* = 0.001) in the jejunum, and although it was not significant, it could still be seen that the LLEP group increased the expression of jejunum MUC2 (*p* = 0.082), and the LLEP group increased the expression of Claudin-2 (*p* < 0.001) in the ileum, while the LEP group elevated the expression of MUC2 (*p* = 0.023) in the ileum.

**Figure 3:**
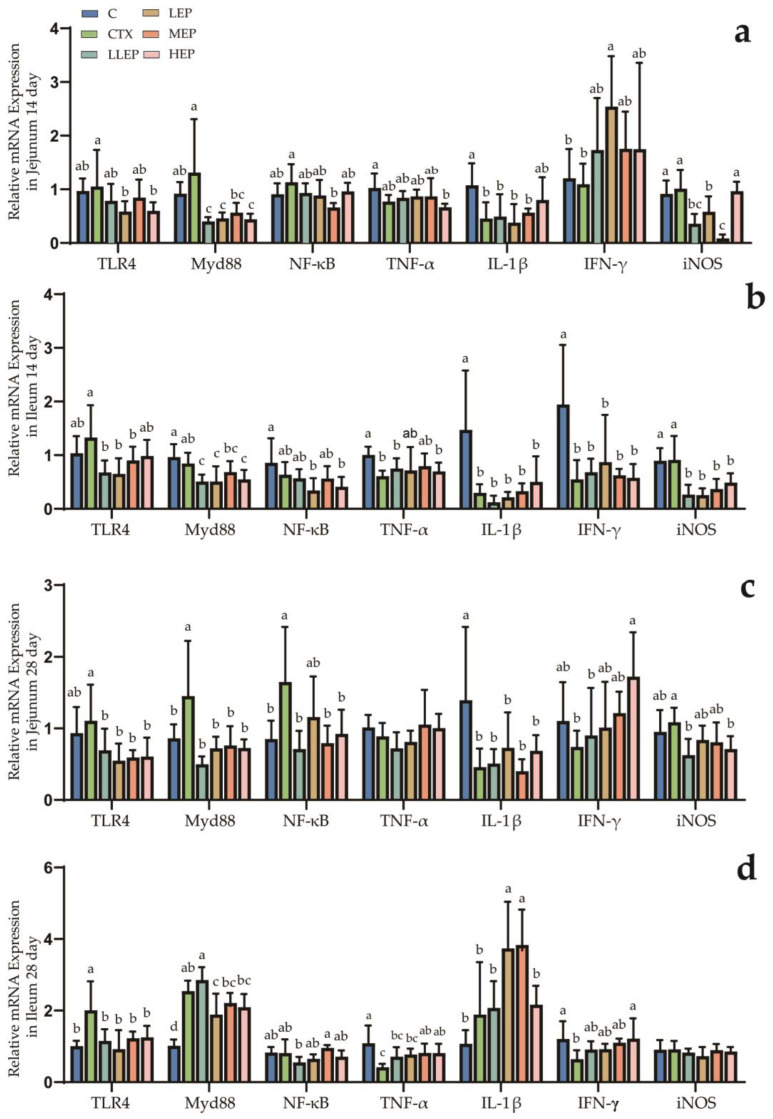
Effect of dietary addition of EPP on the expression of immunity-related genes in the intestinal tract of immunosuppressed chickens. (**a**): Effect of dietary addition of EPP on the expression of immunity-related genes in the jejunum tract of immunosuppressed chickens at 14 days; (**b**): Effect of dietary addition of EPP on the expression of immunity-related genes in the ileum tract of immunosuppressed chickens at 14 days; (**c**): Effect of dietary addition of EPP on the expression of immunity-related genes in the jejunum tract of immunosuppressed chickens at 28 days; (**d**): Effect of dietary addition of EPP on the expression of immunity-related genes in the ileum tract of immunosuppressed chickens at 28 days. ^abcd^ Values within a column followed by different superscripts are significantly different. *p* < 0.05. Abbreviations: C, unimmunosuppressed broilers fed a basic diet; CTX, immunosuppressed broilers fed a basic diet; LLEP, for immunosuppressed broilers, 100 mg/kg of EPP were added to the basic diet.; LEP, for immunosuppressed broilers, 200 mg/kg of EPP were added to the basic diet; MEP, for immunosuppressed broilers, 400 mg/kg of EPP were added to the basic diet; and HEP, for immunosuppressed broilers, 800 mg/kg of EPP were added to the basic diet.

As shown in Figure 3, at 14 days of age, the LEP and HEP groups reduced the expression of TLR4 (*p* < 0.001) in the jejunum. The LLEP, LEP, MEP, and HEP groups all decreased the expression of MyD88 (*p* < 0.001) in the jejunum, with the most significant reductions observed in the LLEP, LEP, and HEP groups. The expression of NF-κB in the jejunum of the MEP group showed a decreasing trend (*p* = 0.073), but not significantly, while the LEP group increased the expression of IFN-γ (*p* = 0.017). Additionally, the LLEP, LEP, and HEP groups decreased the expression of iNOS (*p* = 0.042) in the jejunum. The LLEP group exhibited a decrease in TLR4 (*p* = 0.016) expression in the ileum. Furthermore, the LLEP, LEP, and HE groups demonstrated a reduction in MyD88 (*p* < 0.001) expression, while the LLEP, LEP, MEP, and HEP groups all showed decreased iNOS (*p* < 0.001) expression in the ileum. At 28 days of age, the LLEP, LEP, MEP, and HEP groups all reduced TLR4 (*p* = 0.047) expression in the jejunum. The LLEP, LEP, MEP, and HEP groups also decreased MyD88 (*p* = 0.006) expression, while the LLEP, MEP, and HEP groups reduced NF-κB (*p* = 0.031) expression. The expression of IFN-γ (*p* = 0.098) in the jejunum of the HEP group showed an increasing trend, while that of iNOS (*p* = 0.079) in the jejunum of the LLEP group showed a decreasing trend. The LLEP, LEP, MEP, and HEP groups all significantly reduced TLR4 (*p* = 0.009) expression, while the MEP and HEP groups significantly upregulated IL-1β (*p* < 0.001) expression in the ileum.

**Figure 4:**
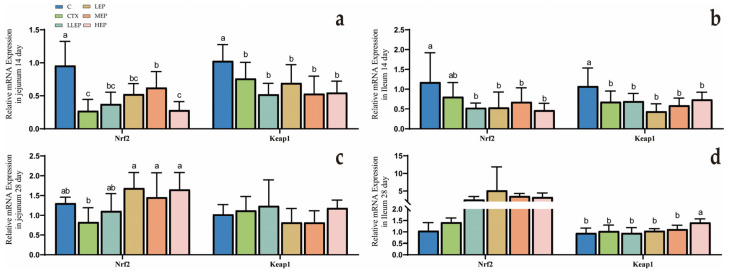
Effect of dietary addition of EPP on the expression of oxidation-related genes in the intestine of immunosuppressed chickens. (**a**): Effect of dietary addition of EPP on the expression of oxidation-related genes in the jejunum of immunosuppressed chickens at 14 days; (**b**): Effect of dietary addition of EPP on the expression of oxidation-related genes in the ileum of immunosuppressed chickens at 14 days; (**c**): Effect of dietary addition of EPP on the expression of oxidation-related genes in the jejunum of immunosuppressed chickens at 28 days; (**d**): Effect of dietary addition of EPP on the expression of oxidation-related genes in the ileum of immunosuppressed chickens at 28 days. ^abc^ Values within a column followed by different superscripts are significantly different. *p* < 0.05. Abbreviations: C, unimmunosuppressed broilers fed a basic diet; CTX, immunosuppressed broilers fed a basic diet; LLEP, for immunosuppressed broilers, 100 mg/kg of EPP were added to the basic diet.; LEP, for immunosuppressed broilers, 200 mg/kg of EPP were added to the basic diet; MEP, for immunosuppressed broilers, 400 mg/kg of EPP were added to the basic diet; and HEP, for immunosuppressed broilers, 800 mg/kg of EPP were added to the basic diet.

As shown in Figure 4, at 14 days of age, the MEP group increased the expression of Nrf2 (*p* < 0.001) in the jejunum. At 28 days of age, the LEP, MEP, and HEP groups all significantly enhanced Nrf2 (*p* = 0.023) expression in the jejunum. The HEP group exhibited a significant increase in Keap1 (*p* = 0.015) expression in the ileum.

### 3.5. Effects of Antioxidants in Immunosuppressed Chickens

**Figure 5:**
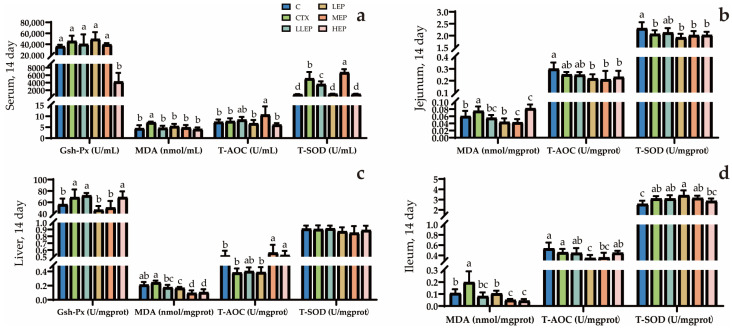
The effect of adding EPP to the diet for 14 days on the antioxidation of immunosuppressed chickens. (**a**): The effect of adding EPP to the diet on the antioxidant capacity of serum in 14 days immunosuppressed chickens; (**b**): The effect of adding EPP to the diet on the antioxidant capacity of jejunum in 14 days immunosuppressed chickens; (**c**): The effect of adding EPP to the diet on the antioxidant capacity of liver in 14 days immunosuppressed chickens; (**d**): The effect of adding EPP to the diet on the antioxidant capacity of ileum in 14 days immunosuppressed chickens. ^abcd^ Values within a column followed by different superscripts are significantly different. *p* < 0.05. Abbreviations: C, unimmunosuppressed broilers fed a basic diet; CTX, immunosuppressed broilers fed a basic diet; LLEP, for immunosuppressed broilers, 100 mg/kg of EPP were added to the basic diet.; LEP, for immunosuppressed broilers, 200 mg/kg of EPP were added to the basic diet; MEP, for immunosuppressed broilers, 400 mg/kg of EPP were added to the basic diet; and HEP, for immunosuppressed broilers, 800 mg/kg of EPP were added to the basic diet.

As shown in Figure 5, at 14 days of age, the LLEP, LEP, MEP, and HEP groups all significantly reduced serum MDA (*p* = 0.025) levels compared to the CTX group. Additionally, the MEP group significantly increased serum T-AOC (*p* = 0.006) and T-SOD (*p* < 0.001) levels compared to the CTX group. The LLEP, LEP, MEP, and HEP groups all significantly reduced liver MDA (*p* < 0.001) levels compared to the CTX group, with the MEP and HEP groups showing the most pronounced effects. Additionally, both the MEP and HEP groups significantly increased liver T-AOC (*p* < 0.001) levels compared to the CTX group. The LLEP, LEP, and MEP groups significantly reduced MDA (*p* < 0.001) levels in the jejunum compared to the CTX group, with the LEP and MEP groups showing the most pronounced effects. The LLEP, LEP, MEP, and HEP groups significantly reduced MDA(*p* < 0.001) levels in the ileum, with the MEP and HEP groups showing the greatest effectiveness.

**Figure 6:**
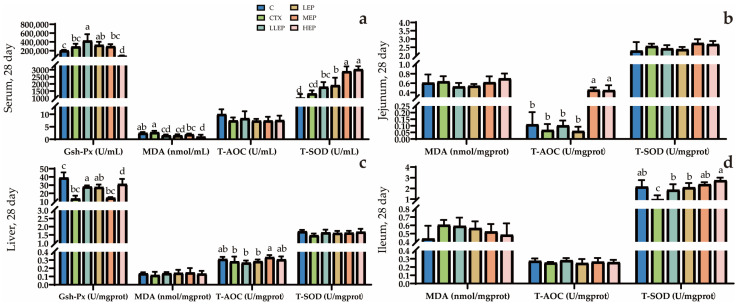
The effect of adding EPP to the diet for 28 days on the antioxidation of immunosuppressed chickens. (**a**): The effect of adding EPP to the diet on the antioxidant capacity of serum in 28 days immunosuppressed chickens; (**b**): The effect of adding EPP to the diet on the antioxidant capacity of jejunum in 28 days immunosuppressed chickens; (**c**): The effect of adding EPP to the diet on the antioxidant capacity of liver in 28 days immunosuppressed chickens; (**d**): The effect of adding EPP to the diet on the antioxidant capacity of ileum in 28 days immunosuppressed chickens. ^abcd^ Values within a column followed by different superscripts are significantly different. *p* < 0.05. Abbreviations: C, unimmunosuppressed broilers fed a basic diet; CTX, immunosuppressed broilers fed a basic diet; LLEP, for immunosuppressed broilers, 100 mg/kg of EPP were added to the basic diet.; LEP, for immunosuppressed broilers, 200 mg/kg of EPP were added to the basic diet; MEP, for immunosuppressed broilers, 400 mg/kg of EPP were added to the basic diet; and HEP, for immunosuppressed broilers, 800 mg/kg of EPP were added to the basic diet.

As shown in Figure 6, at 28 days of age, the LLEP group significantly increased serum GSH-Px (*p* < 0.001) while decreasing MDA (*p* = 0.001) levels compared to the CTX group. The LEP group significantly increased serum T-SOD (*p* < 0.001) content and decreased MDA levels, while the MEP group significantly elevated serum T-SOD levels. The HEP group also significantly enhanced serum T-SOD content compared to the CTX group. The LLEP, LEP, and HEP groups significantly increased liver GSH-Px (*p* < 0.001) content compared to the CTX group. The MEP group significantly enhanced liver T-AOC (*p* = 0.032) levels content compared to the CTX group. Both the MEP and HEP groups significantly increased T-AOC (*p* < 0.001) levels in the jejunum. The LLEP, LEP, MEP, and HEP groups significantly increased T-SOD (*p* < 0.001) levels in the ileum, with the HEP group demonstrating the most pronounced effect.

### 3.6. Effect of Immunosuppressed Chickens on Biochemical Data

As shown in Table 4, at 14 days of age, the MEP group significantly increased serum levels of ALB (*p* < 0.05). At 28 days of age, the LLEP group significantly increased serum levels of ALT, while the LLEP, LEP, and MEP groups significantly increased serum levels of ALB (*p* < 0.05). Furthermore, the LLEP, LEP, MEP, and HEP groups significantly elevated serum levels of TP (*p* < 0.05).

## 4. Discussion

Cyclophosphamide (CTX), a commonly used chemotherapy drug in clinical practice, is widely employed to establish immunosuppression models in experimental research. It can inhibit bone marrow hematopoiesis, reduce lymphocyte proliferation, cytokine secretion, and lower humoral and cellular immune levels [30]. Echinacea, a popular botanical medicine in Europe and the United States, is primarily used to alleviate and prevent upper respiratory tract infections. Echinacea purpurea polysaccharides (EPP), one of its key compounds, have been shown to maintain the functional integrity of the intestinal barrier, in addition to exhibiting strong antioxidant activity [32]. However, the effects of EPP on CTX-induced inflammation, oxidative stress, intestinal barrier damage, and serum biochemistry remain poorly understood. This study therefore aimed to investigate the modulatory effects of EPP on CTX-induced immunosuppression in Mahuang chickens, with a specific focus on growth performance, intestinal barrier integrity, and serum biochemistry.

There were no significant differences in average daily gain (ADG), average daily feed intake (ADFI), and feed-to-gain ratio (F/G) among the groups prior to CTX injection. One week after CTX injection, the ADG of the CTX group was significantly reduced, consistent with the findings of Lu et al. [33]. This decline is likely attributable to CTX-induced impairment of immune function, which likely reduced feeding and nutrient-absorption capacity in the broilers. Supplementation with EPP effectively mitigated this effect, indicating its potential to alleviate CTX-induced immunosuppression. This improvement may be due to the role of EPP in enhancing intestinal absorption, although excessively high doses could potentially cause intestinal damage. Specifically, the LLEP, LEP, and MEP groups showed an increase in ADG, while the LLEP and LEP groups exhibited a decrease in ADFI, and the LLEP, LEP, MEP, and HEP groups displayed a reduction in FCR. Among these, the MEP group demonstrated the most favorable combined effect. The immune organ index, organ-to-body weight ratio, serves as a valuable indicator of immune status in poultry. Key immune organs, such as the spleen, thymus, and bursa, play critical roles in poultry immunity, including disease prevention and resistance to pathogen invasion [25]. A study by Xu et al. [30] reported that CTX significantly reduced the thymus, spleen, and bursa indices in yellow-feathered broilers, resulting in immune organ damage. These results are consistent with the observations recorded on day 14 in the present experiment. Accumulating evidence suggests that plant-derived polysaccharides can enhance immune function by promoting lymphocyte proliferation and modulating immune organ indices. For instance, Li et al. [34] demonstrated that astragalus polysaccharide (APS) could counteract the reduction in immune organ indices caused by CTX. In the present study, supplementation with EPP showed a trend toward improved immune organ indices, with the 800 mg/kg EPP dose yielding the most significant improvement in spleen indices.

The small intestine is critical for nutrient absorption, and the length of the small intestinal villi is positively correlated with the number of epithelial cells, longer villi provide a greater surface area, which enhances the absorption capacity of the intestine [35]. Crypt depth reflects the maturity and proliferation rate of epithelial cells, with shallower crypts indicating enhanced cell maturation and improved nutrient absorption capacity. The ratio of villus height to crypt depth (VH/CD) is a comprehensive indicator of the small intestine’s absorptive capacity [36]. In this study, we observed that supplementation with EPP led to an increase in villus height, a decrease in crypt depth, and an improvement in the VH/CD ratio, thereby mitigating the morphological damage induced by CTX. These findings indicate that EPP contributes to enhanced nutrient absorption. Specifically, 400 mg/kg EPP significantly improved jejunal morphology, 800 mg/kg EPP markedly enhanced duodenal morphology, and both 100 mg/kg and 200 mg/kg EPP significantly improved ileal morphology.

Morphological changes are often accompanied by changes in gut barrier gene expression. Mucin 2 (MUC2) is a high-molecular-weight glycoprotein present in the intestinal mucosa, where it plays a key role in maintaining the integrity of the intestinal barrier [37]. A reduction in MUC2 levels compromises the protective function of the intestinal barrier [38]. In the present study, the decrease in MUC2 levels and alterations in tight junction proteins (e.g., Claudin1, Claudin2, Occludin, and ZO-1) in the jejunum and ileum following CTX injection indicated damage to the intestinal barrier [39]. However, the increased expression of MUC2 and tight junction proteins observed after EPP supplementation suggested that this damage was mitigated. This is the same as the results of intestinal villi. In the CTX group, the villi length decreased, while in the groups with EPP added to the feed, the villi length partially increased. Specifically, supplementation with 200 mg/kg EPP significantly enhanced MUC2 expression in the ileum, while 100 mg/kg, 400 mg/kg, and 800 mg/kg EPP improved the expression of tight junction proteins (e.g., Claudin-1, Claudin-2 and Occludin). Intestinal immunity plays a vital role in maintaining various physiological functions in animals. Interleukin-1 beta (IL-1β) and interferon-gamma (IFN-γ) are key cytokines involved in immune responses. IL-1β promotes the activation of inflammatory cells in response to pathogen invasion, while IFN-γ enhances cellular immunity by activating macrophages and supporting their antigen-presenting ability [40]. Toll-like receptor 4 (TLR4) is a crucial pattern recognition receptor (PRR) on innate immune cells, triggering the production of inflammatory cytokines in response to infection or tissue damage. MyD88, a TLR4 adaptor protein, plays a central role in initiating inflammatory signaling upon ligand binding [41]. In this study, we observed that EPP reduced the expression of TLR4, MyD88, and iNOS, while increasing IFN-γ levels in both the jejunum and ileum. These results suggest that EPP may have a restorative effect on intestinal immunity. Notably, 200 mg/kg EPP significantly reduced TLR4, MyD88, and iNOS expression in the jejunum, whereas 400 mg/kg EPP significantly decreased TLR4 and iNOS expression and increased IL-1β and IFN-γ levels in the ileum. The intestine is a vital organ for nutrient absorption in poultry [42], and improvements in intestinal immunity are consequently reflected in growth performance. These findings are consistent with the growth performance results, suggesting that EPP may alleviate the immunosuppressive effects induced by CTX.

Keap1 and Nrf2 are critical regulators of the cellular response to oxidative stress. Upon oxidative stress, reactive cysteine residues in Keap1 are modified, which prevents the ubiquitination of Nrf2, stabilizing it and facilitating its translocation to the nucleus, where it activates the expression of cytoprotective antioxidant enzymes [43]. As a key transcription factor, Nrf2 mediates the maintenance of cellular homeostasis and adaptive stress responses [44]. The antioxidant activity of plant polysaccharides has been well documented [45], and a study by Xin et al. [46] confirmed that certain polysaccharides alleviate oxidative stress through the Keap1/Nrf2 pathway. In the present experiment, supplementation with 400 mg/kg EPP increased Nrf2 expression in the jejunum, while 800 mg/kg EPP significantly enhanced Keap1 expression in the ileum.

The intestine is not only essential for digesting and absorbing nutrients but also represents a major site vulnerable to free radical attack in animals [47]. The assessment of antioxidant markers such as glutathione peroxidase (GSH-Px), malondialdehyde (MDA), total antioxidant capacity (T-AOC), and superoxide dismutase (T-SOD) provides an evaluation of immune stress-induced damage [48]. Oxidative stress reflects an imbalance between the production of oxidants and antioxidants, where an excess of reactive oxygen species (ROS) can inhibit antioxidant enzyme activity, including T-SOD, and deplete GSH-Px levels [49]. GSH-Px, an antioxidant enzyme present in cells, plays a critical role in cellular metabolism and defense against oxidative stress [50]. In this study, supplementation with EPP increased GSH-Px levels in both liver and serum, suggesting that EPP enhance the oxidative defense capacity of these tissues. Previous studies have demonstrated that CTX injection leads to elevated MDA levels and decreased T-AOC and T-SOD activity, which is consistent with the findings of the present experiment [51,52]. However, the EPP-supplemented groups showed reduced MDA concentrations and increased T-AOC and T-SOD activities in the serum, liver, and small intestine. Overall, all EPP supplementation groups were effective in reducing MDA content, with 100 mg/kg EPP being most effective in increasing GSH-Px levels in serum and liver. In contrast, 400 mg/kg EPP was most effective in enhancing T-AOC levels in the serum, liver, and small intestine.

Serum biochemical indices serve as important indicators of an animal’s health status and metabolic function [53]. Total protein (TP), which comprises albumin (ALB) and globulin (GLB), reflects the efficiency of protein absorption and metabolism. An increase in TP levels, within the normal range, indicates enhanced metabolic, digestive, and absorptive capacities of the animal [54].

In the present study, supplementation with 400 mg/kg EPP increased ALB levels. Additionally, TP levels were elevated in all EPP treatment groups. These results suggest that EPP supplementation improves liver function and enhances nutrient absorption in immunosuppressed chickens, thereby supporting their growth and development.

Based on a comprehensive analysis of the overall experimental results, the supplementation levels of 400 mg/kg and 800 mg/kg Echinacea polysaccharides demonstrated the most favorable effects. Both dosages significantly improved growth performance, intestinal morphology, the expression of tight junction genes, certain inflammatory and oxidation-related genes, as well as antioxidant indicators. However, no statistically significant differences were observed between the 800 mg/kg and 400 mg/kg groups in terms of these improvements. Taking supplementation costs into consideration, 400 mg/kg is identified as the optimal dosage.

Although this study primarily focused on Mahuang chickens, the findings offer valuable insights into the effects of EPP on growth performance, intestinal barrier integrity, immune function, antioxidant activity, and biochemical parameters in other chicken breeds. While physiological characteristics may vary among different broiler varieties, the optimal dosage identified in this study can still serve as a useful reference for future production applications. It should be noted that this research only partially elucidated the effects of EPP, and the underlying mechanisms remain unclear. Further studies are warranted to fully unravel these mechanisms.

## 5. Conclusions

For the intestinal barrier, all four doses of EPP improved intestinal morphology and increased intestinal-barrier mRNA expression, which may be related to the improved intestinal absorption of the polysaccharides. In addition, EPP promoted antioxidant vigor and immunity in broilers. Overall, supplementing the diet with EPP created a healthy intestinal environment and improved feed utilization. The best results were obtained by adding 400 mg/kg of EPP to the diet.

## Figures and Tables

**Table 1:** Effect of EPP on growth performance of immunosuppressed broilers.

Item ^1^	C	CTX	LLEP	LEP	MEP	HEP	*p*-Value
ADG							
7 days old	6.27 ± 0.24	6.37 ± 0.54	6.14 ± 0.28	6.74 ± 0.28	6.40 ± 0.15	6.52 ± 0.84	0.308
14 days old	10.16 ± 0.19 ^a^	7.44 ± 0.45 ^b^	7.29 ± 1.66 ^b^	6.98 ± 0.76 ^b^	7.31 ± 0.35 ^a^	7.70 ± 0.29 ^b^	<0.001
21 days old	11.57 ± 0.60 ^a^	9.45 ± 1.07 ^b^	10.48 ± 1.45 ^ab^	11.21 ± 0.46 ^a^	11.46 ± 0.62 ^a^	11.41 ± 0.72 ^a^	0.002
28 days old	14.64 ± 0.40	12.49 ± 1.13	13.56 ± 1.75	13.10 ± 1.83	13.57 ± 2.07	14.00 ± 0.98	0.221
ADFI							
7 days old	7.65 ± 1.04	9.50 ± 3.06	9.10 ± 1.53	7.35 ± 1.81	7.30 ± 1.86	7.85 ± 1.15	0.212
14 days old	15.60 ± 0.11 ^ab^	16.15 ± 4.66 ^a^	15.40 ± 2.41 ^ab^	12.05 ± 0.60 ^c^	12.80 ± 1.10 ^bc^	13.40 ± 0.22 ^abc^	0.012
21 days old	24.35 ± 0.05 ^c^	28.50 ± 3.72 ^a^	27.45 ± 2.46 ^ab^	24.45 ± 1.48 ^c^	25.55 ± 0.93 ^bc^	24.90 ± 1.75 ^bc^	0.006
28 days old	31.05 ± 0.27 ^ab^	34.15 ± 3.56 ^a^	33.35 ± 1.91 ^ab^	29.60 ± 1.31 ^b^	31.25 ± 0.60 ^bc^	29.05 ± 5.09 ^bc^	0.018
F/G							
7 days old	1.23 ± 0.17	1.48 ± 0.47	1.48 ± 0.26	1.10 ± 0.28	1.14 ± 0.30	1.21 ± 0.20	0.118
14 days old	1.54 ± 0.03 ^b^	2.18 ± 0.67 ^a^	2.19 ± 0.52 ^a^	1.75 ± 0.20 ^ab^	1.76 ± 0.21 ^ab^	1.75 ± 0.07 ^ab^	0.021
21 days old	2.11 ± 0.11 ^c^	3.04 ± 0.42 ^a^	2.67 ± 0.47 ^b^	2.118 ± 0.08 ^c^	2.24 ± 0.14 ^c^	2.19 ± 0.18 ^c^	<0.001
28 days old	2.12 ± 0.05 ^bc^	2.75 ± 0.27 ^a^	2.49 ± 0.33 ^b^	2.30 ± 0.36 ^bc^	2.35 ± 0.39 ^bc^	2.08 ± 0.36 ^c^	0.010

^abc^ Values within a column followed by different superscripts are significantly different. *p* < 0.05; ^1^ ADG, average daily gain; ADFI, average daily feed intake; F/G, feed conversion ratio. Abbreviations: C, unimmunosuppressed broilers fed a basic diet; CTX, immunosuppressed broilers fed a basic diet; LLEP, for immunosuppressed broilers, 100 mg/kg of EPP were added to the basic diet.; LEP, for immunosuppressed broilers, 200 mg/kg of EPP were added to the basic diet; MEP, for immunosuppressed broilers, 400 mg/kg of EPP were added to the basic diet; and HEP, for immunosuppressed broilers, 800 mg/kg of EPP were added to the basic diet.

**Table 2:** Effect of Echinacea purpurea polysaccharides on organ index in immunosuppressed chickens.

Item	C	CTX	LLEP	LEP	MEP	HEP	*p*-Value
14 days old							
Splenic index	0.10 ± 0.02 ^a^	0.07 ± 0.03 ^bc^	0.06 ± 0.02 ^c^	0.07 ± 0.02 ^abc^	0.08 ± 0.02 ^abc^	0.09 ± 0.01 ^ab^	0.026
Thymic index	0.34 ± 0.08 ^a^	0.08 ± 0.05 ^b^	0.15 ± 0.09 ^b^	0.12 ± 0.04 ^b^	0.14 ± 0.08 ^b^	0.08 ± 0.02 ^b^	<0.001
Bursal index	0.38 ± 0.06 ^a^	0.17 ± 0.09 ^c^	0.22 ± 0.10 ^bc^	0.20 ± 0.06 ^bc^	0.20 ± 0.11 ^bc^	0.31 ± 0.14 ^ab^	0.006
28 days old							
Splenic index	0.11 ± 0.03	0.12 ± 0.05	0.12 ± 0.03	0.11 ± 0.02	0.10 ± 0.04	0.10 ± 0.04	0.995
Thymic index	0.39 ± 0.20 ^a^	0.14 ± 0.07 ^b^	0.11 ± 0.08 ^b^	0.14 ± 0.06 ^b^	0.14 ± 0.09 ^b^	0.12 ± 0.05 ^b^	0.002
Bursal index	0.44 ± 0.11	0.57 ± 0.20	0.57 ± 0.18	0.59 ± 0.12	0.68 ± 0.12	0.61 ± 0.24	0.283

^abc^ Values within a column followed by different superscripts are significantly different. *p* < 0.05. Abbreviations: C, unimmunosuppressed broilers fed a basic diet; CTX, immunosuppressed broilers fed a basic diet; LLEP, for immunosuppressed broilers, 100 mg/kg of EPP were added to the basic diet.; LEP, for immunosuppressed broilers, 200 mg/kg of EPP were added to the basic diet; MEP, for immunosuppressed broilers, 400 mg/kg of EPP were added to the basic diet; and HEP, for immunosuppressed broilers, 800 mg/kg of EPP were added to the basic diet.

**Table 3:** Effect of immunosuppressed chickens on intestinal morphology.

Item ^1^	C	CTX	LLEP	LEP	MEP	HEP	*p*-Value
14 days old							
Jejunum							
VH (μm)	748.68 ± 40.02 ^a^	688.53 ± 17.68 ^b^	807.76 ± 31.48 ^ab^	737.43 ± 51.16 ^ab^	804.29 ± 56.62 ^a^	731.86 ± 36.35 ^ab^	0.028
CD (μm)	146.35 ± 16.81 ^b^	158.08 ± 7.05 ^ab^	164.81 ± 3.64 ^a^	152.33 ± 4.82 ^ab^	155.76 ± 3.57 ^ab^	127.86 ± 6.59 ^c^	0.003
VH/CD	5.58 ± 1.47	4.50 ± 0.72	4.91 ± 0.33	4.84 ± 0.71	5.17 ± 0.72	5.89 ± 1.16	0.137
Ileum							
VH (μm)	620.40 ± 51.88 ^b^	600.23 ± 28.19 ^b^	657.30 ± 57.85 ^ab^	701.22 ± 6.67 ^a^	637.78 ± 28.85 ^b^	642.53 ± 16.29 ^ab^	0.037
CD (μm)	154.23 ± 23.60	162.40 ± 10.20	163.01 ± 8.54	163.04 ± 5.29	164.57 ± 7.53	158.37 ± 2.54	0.885
VH/CD	4.29 ± 1.03	3.72 ± 0.40	4.03 ± 0.38	4.26 ± 0.36	3.89 ± 0.37	4.06 ± 0.24	0.422
28 days old							
Jejunum							
VH (μm)	756.77 ± 37.11 ^c^	711.08 ± 8.29 ^c^	902.78 ± 6.23 ^b^	974.20 ± 1.51 ^a^	990.53 ± 12.78 ^a^	891.96 ± 23.39 ^b^	<0.001
CD (μm)	129.47 ± 15.70 ^b^	125.92 ± 19.58 ^b^	126.50 ± 8.37 ^b^	143.67 ± 16.78 ^ab^	143.01 ± 7.82 ^ab^	183.17 ± 9.46 ^a^	0.016
VH/CD	4.79 ± 1.17	3.63 ± 0.62	4.98 ± 1.11	5.08 ± 2.09	4.09 ± 0.24	4.38 ± 0.15	0.205
Ileum							
VH (μm)	650.19 ± 20.99 ^bc^	614.84 ± 26.55 ^c^	756.11 ± 57.57 ^a^	727.05 ± 16.00 ^ab^	649.37 ± 14.25 ^bc^	657.99 ± 19.07 ^bc^	0.017
CD (μm)	139.95 ± 4.93	173.38 ± 10.87	152.28 ± 5.11	149.16 ± 10.10	156.83 ± 5.13	151.82 ± 6.55	0.086
VH/CD	5.83 ± 1.04	5.99 ± 1.50	7.23 ± 1.04	6.99 ± 1.08	7.61 ± 2.00	5.95 ± 2.12	0.213

^abc^ Values within a column followed by different superscripts are significantly different. *p* < 0.05. ^1^ VH, intestinal villus length; CD, intestinal crypt depth; VH/CD, ratio of villus length to crypt depth. Abbreviations: C, unimmunosuppressed broilers fed a basic diet; CTX, immunosuppressed broilers fed a basic diet; LLEP, for immunosuppressed broilers, 100 mg/kg of EPP were added to the basic diet.; LEP, for immunosuppressed broilers, 200 mg/kg of EPP were added to the basic diet; MEP, for immunosuppressed broilers, 400 mg/kg of EPP were added to the basic diet; and HEP, for immunosuppressed broilers, 800 mg/kg of EPP were added to the basic diet.

**Table 4:** Effect of EPP on serum biochemistry of immunosuppressed chickens.

Item ^1^	C	CTX	LLEP	LEP	MEP	HEP	*p*-Value
14 days old							
ALB	9.93 ± 0.97 ^b^	10.62 ± 1.07 ^b^	11.32 ± 0.91 ^ab^	11.28 ± 1.33 ^ab^	12.30 ± 1.88 ^a^	10.66 ± 1.48 ^b^	0.037
TP	24.70 ± 2.48	23.38 ± 2.92	26.50 ± 1.98	25.93 ± 2.93	27.83 ± 3.59	24.81 ± 2.99	0.107
GLB	14.75 ± 1.75	13.33 ± 1.67	15.17 ± 1.17	14.75 ± 1.75	15.57 ± 1.90	14.25 ± 1.58	0.227
28 days old							
ALB	8.58 ± 0.86 ^b^	8.05 ± 1.80 ^b^	10.55 ± 1.87 ^a^	10.67 ± 1.77 ^a^	10.83 ± 0.59 ^a^	9.15 ± 0.66 ^ab^	0.012
TP	21.85 ± 2.24 ^bc^	20.80 ± 4.09 ^c^	26.82 ± 3.760 ^ab^	26.60 ± 4.31 ^ab^	28.00 ± 2.75 ^a^	26.12 ± 4.82 ^ab^	0.033
GLB	13.33 ± 1.86	12.75 ± 2.36	16.33 ± 2.07	15.83 ± 2.93	17.25 ± 2.87	16.17 ± 4.58	0.157

^abc^ Values within a column followed by different superscripts are significantly different. *p* < 0.05. ^1^ Total protein (TP), albumin (ALB) and globulin (GLB). Abbreviations: C, unimmunosuppressed broilers fed a basic diet; CTX, immunosuppressed broilers fed a basic diet; LLEP, for immunosuppressed broilers, 100 mg/kg of EPP were added to the basic diet.; LEP, for immunosuppressed broilers, 200 mg/kg of EPP were added to the basic diet; MEP, for immunosuppressed broilers, 400 mg/kg of EPP were added to the basic diet; and HEP, for immunosuppressed broilers, 800 mg/kg of EPP were added to the basic diet.

## Data Availability

The data presented in this study are available upon request from the corresponding author.

## Supplementary Materials

The following supporting information can be downloaded at: https://www.mdpi.com/article/10.3390/ani15203036/s1, Table S1: Nutritional levels of basic dietary formulations for test animals; Table S2: Primer information.
