# Supplementary material for: Effects of Echinacea Purpurea Polysaccharides on Growth Performance, Serum Biochemistry, and Intestinal Health of Immunosuppressed Broilers
Source: Animals (Basel). 2025 Oct 20;15(20):3036. doi: 10.3390/ani15203036 (PMC12562094; doi:10.3390/ani15203036)
Supplement: Supplementary file 1 [file animals-15-03036-s001.zip › animals-3918135-supplementary.pdf]

Table S1. Nutritional levels of basic dietary formulations for test animals, %

Componente	Element
Ingredient (%)	
Corn	54.5
Soybeanmeal (CP, 43%)	33
Fish meal	1
Wheat bran	5.5
Soy oil	1.5
Rapeseed meal	0
Limestone powder	1.4
$CaHPO_4$	1.1
Premix[1]	2
Total	100
Nutrient levels (%)[2]	
Crude protein	21
Calcium	1.05
Sodium	0.16
Chlorine, %	0.16
Total phosphorus	0.5
Lysine	1.2
Methionine	0.55
Threonine	0.19
Metabolizable energy, kcal/kg	2920.58

[1]The premix provided the following per kilogram of diet: VA, 7500 IU; VB_1, 3.5 mg; VB_2, 3.2 mg; VB_5, 22 mg; VB_6, 3.2 mg; VB_{12}, 0.02 mg; VD_3, 1500 IU, VK_3, 2 mg; biotin, 0.12 mg; folic acid, 1.0 mg; pantothenic acid, 28.0 mg; nicotinic acid, 18 mg; antioxidant, 100 mg; Cu, 6.5 mg: Fe, 70mg:Mn, 60 mg: Zn, 60 mg: Se, 0.4 mg.
[2]Metabolizable energy was a calculated value, while the others were measured values.

Table S2. Primer information

Target Gene	Primer Sequence	GeneBank
β-actin	Forward primer : GAGAAATTGTGCGTGACATCA<br>Reverse primer : CCTGAACCTCTCATTGCCA	NM_205518.2
IL-1β	Forward primer : GAAGTGCTTCGTGCTGGAGT<br>Reverse primer : ACTGGCATCTGCCCAGTTC	XM_015297469.3
TNF-α	Forward primer : AATTTGCAGGCTGTTTCTGC<br>Reverse primer : TATGAAGGTGGTGCAGATGG	XM_040647304.2
Claudin-1	Forward primer : GAGGATGACCAGGTCAAGAAG<br>Reverse primer : TGCCCAGCCAATGAAGAG	NM_001013611.2
Nrf2	Forward primer : ATCACCTCTTCTGCACCGAA<br>Reverse primer : GCTTTCTCCCGCTCTTTCTG	XM_046921122.1
TLR4	Forward primer : AGTCTGAAATTGCTGAGCTCAAAT<br>Reverse primer : GCGACGTTAAGCCATGGAAG	NM_001030693.2
ZO-1	Forward primer : CCAAAGACAGCAGGAGGAGA<br>Reverse primer : TGGCTAGTTTCTCTCGTGCA	XM_046925214.1

Occludin	Forward primer : TCATCCTGCTCTGCCTCATCT<br>Reverse primer : CATCCGCCACGTTCTTCAC	XM_046904540.1
MUC2	Forward primer : CATTCAACGAGGAGAGCTGC<br>Reverse primer : TTCCTTGCAGCAGGAACAAC	XM_040673077.2
IFN-γ	Forward primer : GGACATGGCTCCCACACTAC<br>Reverse primer : TGAAGAGGTGCTGAAGGATG	FJ977575.1
Claudin-2	Forward primer : CACCGTCTTCAATCAGGGCT<br>Reverse primer : AGCTGAACTCACTCTTGGGC	NM_001277622.1
Myd88	Forward primer : AGCATTACCAGGGCTGAGTT<br>Reverse primer : TGGTACCATGCCAGCAGTTA	XM_046910878.1
NF-κB	Forward primer : CAGCCCATCTATGACAACCG<br>Reverse primer : TCAGCCCAGAAACGAACCTC	NM_001396038.1
Keap1	Forward primer : CATCGGCATCGCCAACTT<br>Reverse primer : TGAAGAACTCCTCCTGCTTGGA	MN416132.1
iNOS	Forward primer : CCTGTACTGAAGGTGGCTATTGG<br>Reverse primer : AGGCCTGTGAGAGTGTGCAA	D85422.1
